# Unveiling the Pathogenesis of Adenomyosis through Animal Models

**DOI:** 10.3390/jcm11061744

**Published:** 2022-03-21

**Authors:** Xi Wang, Giuseppe Benagiano, Xishi Liu, Sun-Wei Guo

**Affiliations:** 1 Shanghai Obstetrics and Gynecology Hospital, Fudan University, Shanghai 200011, China; wangxi9508@163.com (X.W.); lxsdoc@hotmail.com (X.L.); 2 Faculty of Medicine and Dentistry, Sapienza, University of Rome, 00161 Rome, Italy; pinoingeneva@bluewin.ch; 3 Shanghai Key Laboratory of Female Reproductive Endocrine-Related Diseases, Fudan University, Shanghai 200011, China

**Keywords:** adenomyosis, animal models, endometrial–myometrial interface disruption, falsifiability, pathogenesis, predictivity

## Abstract

Background: Adenomyosis is a common gynecological disorder traditionally viewed as “elusive”. Several excellent review papers have been published fairly recently on its pathogenesis, and several theories have been proposed. However, the falsifiability, explanatory power, and predictivity of these theories are often overlooked. Since adenomyosis can occur spontaneously in rodents and many other species, the animal models may help us unveil the pathogenesis of adenomyosis. This review critically tallies experimentally induced models published so far, with a particular focus on their relevance to epidemiological findings, their possible mechanisms of action, and their explanatory and predictive power. Methods: PubMed was exhaustively searched using the phrase “adenomyosis and animal model”, “adenomyosis and experimental model”, “adenomyosis and mouse”, and “adenomyosis and rat”, and the resultant papers were retrieved, carefully read, and the resultant information distilled. All the retrieved papers were then reviewed in a narrative manner. Results: Among all published animal models of adenomyosis, the mouse model of adenomyosis induced by endometrial–myometrial interface disruption (EMID) seems to satisfy the requirements of falsifiability and has the predictive capability and also Hill’s causality criteria. Other theories only partially satisfy Hill’s criteria of causality. In particular, animal models of adenomyosis induced by hyperestrogenism, hyperprolactinemia, or long-term exposure to progestogens without much epidemiological documentation and adenomyosis is usually not the exclusive uterine pathology consequent to those induction procedures. Regardless, uterine disruption appears to be a necessary but not sufficient condition for causing adenomyosis. Conclusions: EMID is, however, unlikely the sole cause for adenomyosis. Future studies, including animal studies, are warranted to understand how and why in utero and/or prenatal exposure to elevated levels of estrogen or estrogenic compounds increases the risk of developing adenomyosis in adulthood, to elucidate whether prolactin plays any role in its pathogenesis, and to identify sufficient condition(s) that cause adenomyosis.

## 1. Introduction

Adenomyosis, defined as the invasion of the endometrium into the myometrium [1], has been traditionally labeled an “elusive” disease [1,2] because, until the advent of imaging technology, a diagnosis could only be made on a hysterectomy sample, thereby eluding clinical scrutiny, since only advanced stages of the condition were investigated. Even today, it is still viewed as “a riddle, wrapped in mystery, inside an enigma” [3].

“Elusive” also meant a lack of information on the incidence of its occurrence: in the 1990s, its frequency was estimated at between 10% and 80% of women of reproductive age undergoing hysterectomy [4]. Such a wide variation could not be explained by differences in factors known to correlate with the incidence of adenomyosis; rather, it suggested that adenomyosis was often over-diagnosed in surgical specimens and over-represented in older parous or perimenopausal women. 

This unsatisfactory situation changed dramatically when imaging technology, such as ultrasonography and magnetic resonance, was applied to the study of the disease, allowing a non-invasive diagnosis [5,6]. As a consequence of this development, work was intensified on clinical aspects, showing that approximately one-third of women with adenomyosis are asymptomatic [7]. In addition, it was found that in symptomatic patients, the disease manifests itself with abnormal uterine bleeding, pelvic pain, infertility, and progressive dysmenorrhea [8], impacting negatively on the quality of life of the afflicted woman [9]. Adenomyosis is also associated with pregnancy complications, such as increased risk of spontaneous abortions, preterm delivery, and the need for cesarean sections [10,11]. Overall, despite its apparent high prevalence, currently, its clinical management is still a challenge, largely due to its poorly understood pathogenesis and pathophysiology [12].

The epidemiology of adenomyosis has also been investigated, and a few excellent review articles have been published [13,14]. While several risk factors have been identified, it seems that there are always conflicting results, due likely to the various biases in adenomyosis epidemiological studies [14]. These risk factors include earlier age at menarche, spontaneous abortion, induced abortion, evacuation, dilatation and curettage (D&C), and other uterine surgeries [14]. 

**Figure 1:**
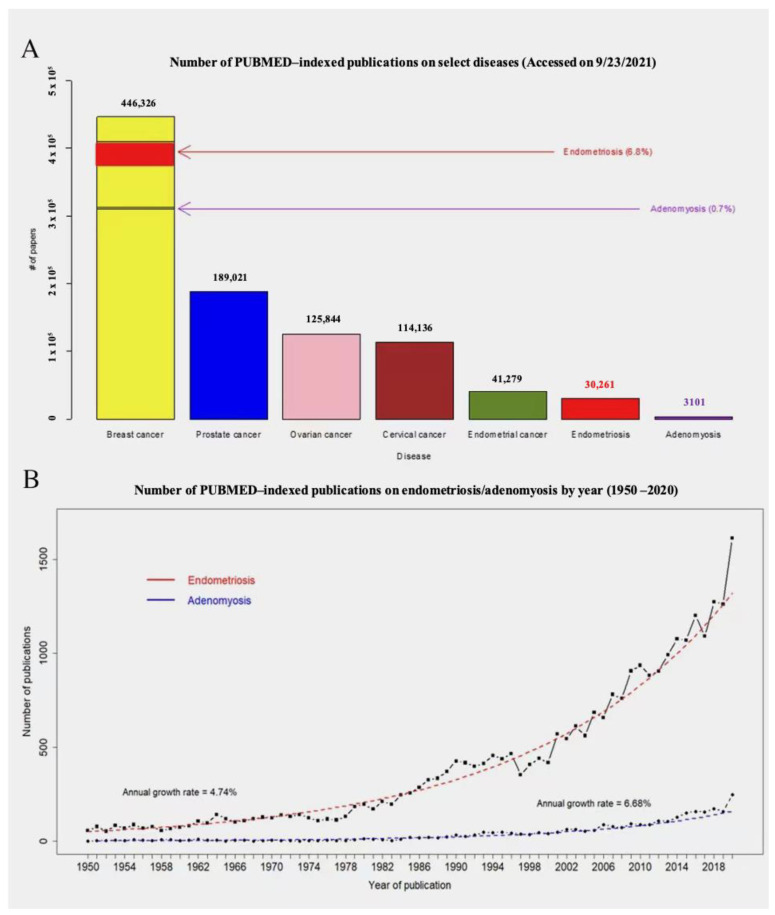
(**A**) A comparison of the number of PubMed-indexed publications on breast cancer, prostate cancer, ovarian cancer, cervical cancer, endometrial cancer, endometriosis, and adenomyosis (accessed on 9 October 2021). The numbers listed on each bar are the exact numbers of publications. (**B**) The numbers of PubMed-indexed publications on endometriosis and adenomyosis in the last 70 years (1950–2020). The dashed curves are the fitted regression curves.

In spite of promising advances, adenomyosis is, overall, an under-researched disease. This can be seen from the number of PubMed-indexed papers on this disease, which is approximately 3100, just a paltry one-tenth of those on endometriosis as of now (Figure 1A) and less than 1% of those on breast cancer. 

Despite this pathetical number of publications, there has been a steady and substantial growth in PubMed-indexed papers on adenomyosis (Figure 1B). In fact, several excellent review papers have been published fairly recently on the pathogenesis of the disease [15,16,17,18,19,20,21,22]. 

In this respect, to improve our understanding of the pathogenesis of adenomyosis, over the years, many animal models have been reported. While adenomyosis can and do occur spontaneously in rodents, such as CD-1 mice [23], SHN and SLN strains [24], GR/A strain [24], C3H/He strain [24], and SMXA recombinant inbred mice [25], it also occurs spontaneously in other species such as rhesus monkeys (*Macaca mulatta*) [26], cats [27], dogs [28], baboons [29], and chimpanzees [30]. It also can be successfully induced in an array of murine models via various procedures. These experimentally induced adenomyosis models offer a consistent, reproducible, and controlled alternative to the spontaneous models and, in many cases, are also much less time-consuming and more economical. There are several excellent reviews on this topic [23,31,32].

In the present review, we first discuss what is known about the pathogenesis of adenomyosis; we then critically tally experimentally induced models that have been published so far, with a particular focus on whether they meet the requirements of falsifiability and predictive capability. After all, a good theory in biomedicine needs to satisfy at least three requirements: falsifiability, explanatory power (i.e., it can explain most, if not all, existing data), and predictive capability. Indeed, if a theory cannot be proven or refuted by experimentation, it is akin to religion, and it would be in need of more theories. In addition, it is not of much utility if it cannot make any useful predictions or serve as a guide for new discoveries or devising novel therapeutics or preventive measures.

Since adenomyosis can also occur spontaneously in many animal species as in humans, it is fair to assume that the mechanism of action in inducing adenomyosis in animals could provide us with a much-needed insight into the pathogenesis of the disease. As such, the resultant hypotheses/theories should be scrutinized by Hill’s criteria that were originally used to gauge the credibility or strength of a causal relationship between disease and a putative risk factor [33]. We also discuss the pros and cons of these models in the light of our current knowledge on pathogenesis and pathophysiology and, finally, identify existing knowledge gaps in the pathogenesis of adenomyosis.

## 2. Methods

PubMed was exhaustively searched using the phrase “adenomyosis and animal model”, “adenomyosis and experimental model”, “adenomyosis and mouse”, and “adenomyosis and rat”, and the resultant papers, in English and published from 1 January 1950 to 31 January 2022, were retrieved, carefully read, and the resultant information distilled. All the retrieved papers were then reviewed in a narrative manner.

## 3. Results

### 3.1. Pathogenetic Hypotheses and Theories

Currently, there are two widely accepted theories on the pathogenesis of adenomyosis, namely, metaplasia and invagination [18,19,21]. The metaplasia theory postulates that the endometrial cells in the muscular layer originate from the metaplasia of Müllerian remnants or stem cells [16,34,35]. In contrast, the invagination theory stipulates that the direct invasion of the endometrium to the muscle layer results from the process of tissue injury and repair that leads to the formation of lesions [36,37,38]. One important building block within the invagination theory hinges on the tissue injury and repair (TIAR) hypothesis, proposed by Leyendecker and his associates [36,37]. Unfortunately, so far, there have been no experimental data to support or refute either of the theories or the TIAR hypothesis [39].

A somewhat related postulation regarding the pathogenesis of adenomyosis is the stem cell hypothesis [16,34]. As recently summarized [40], three main types of endogenous endometrial adult stem cells, namely, stromal, epithelial progenitor, and endothelial, have been identified in the endometrium in addition to bone-marrow-derived stem cells. This hypothesis posits that individual progenitor cells or stem cells, possibly bone marrow derived, may disseminate locally or spread through the circulation to establish the initial focus of adenomyotic lesions if deposited in the myometrium or the endometrial–myometrial interface (EMI). This deposition may result from local trauma or injury since the injured tissues are likely to cause aggregation of platelets, which may recruit the stem cells [41] and induce hypoxia [42], increased estrogen production [43], and hypoxia-induced inflammation [44]. However, it is unclear as to why and how this injury—the apparent primum movens—occurs. It is equally unclear how these recruited stem cells are turned into endometrial epithelial and stromal cells that respond to hormonal fluctuations and undergo cyclic bleeding and repair.

Some subtypes of adenomyosis are reported to be closely linked, in terms of both biological characteristics and pathogenesis, with deep endometriosis [45,46,47,48,49,50], raising the prospect that the adenomyotic lesions may originate from the neighboring deep endometriotic lesions. However, in the absence of any data delineating the phylogenetic relationship between the adenomyotic and deep endometriotic lesions, it is equally plausible that the deep endometriotic lesions may originate from adenomyotic foci near to the serosa or that they could arise rather independently [51].

One fundamental requirement for any good theory or hypothesis in biomedicine is falsifiability. For ethical reasons, to prove or disprove any theory on adenomyosis pathogenesis by human experimentation is likely to be out of the question unless long-term prospective epidemiological studies can be feasibly carried out. This, in turn, will depend on agreeing on the criteria for a non-invasive diagnostic method. In other words, the challenge in pathogenesis research on adenomyosis is to establish a non-invasive yet definitive diagnosis, especially for early stage of adenomyosis, which so far is lacking. In this regard, the use of animal experimentation can be of great help. This is especially true since, unlike endometriosis, adenomyosis can and does occur spontaneously in many animal species, including, but not limited to, rodents [23,32]. Therefore, animal models of adenomyosis may provide a practical means to unveil the pathogenesis of adenomyosis.

### 3.2. The Quest for the Primum Movens

In exploring the pathogenesis of adenomyosis, the issue of its primum movens, or the first hit, is of vital importance. This is because once the lesion is established, presumably it would undergo cyclic bleeding just as the eutopic endometrium [52,53] and, as such, effectively become a wound that undergoes repeated tissue injury and repair (ReTIAR) [54], progressing to fibrosis through epithelial–mesenchymal transition (EMT), fibroblast-to-myofibroblast transdifferentiation (FMT), and smooth muscle metaplasia (SMM) [55,56]. In other words, once the primum movens is effectively applied, everything will set in motion, more or less on its own, resulting in adenomyosis as we see it.

Of course, the budding lesion may still be subject to removal by immune cells, and the successful establishment of an adenomyotic lesion may well be the end result of this tug of war between constant seeding and elimination. However, once the lesion is well established, and barring any extraneous factors or behavioral/lifestyle changes that either boost the immune function or slow down the progression (such as caloric restriction or eustress [57,58]), the established lesion will progress. Conceivably, the lesional progression could, in fact, be facilitated by psychogenic stress [59,60], high-fat diet [61], lower dairy consumption [62], surgery [63,64], and history of adverse early life events [65], and perhaps other factors yet to be identified.

### 3.3. Animal Models of Adenomyosis

**Figure 2:**
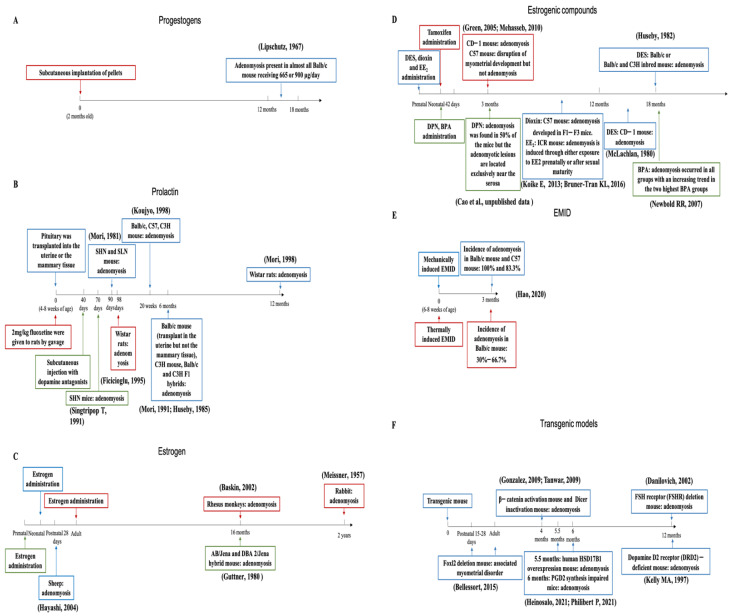
Timelines of all animal adenomyosis models by species, strain, induction method, duration of induction, and results. Panels (**A**–**F**) represented different inducers. (**A**) Progestogens; (**B**) Prolactin; (**C**) Estrogen; (**D**) Estrogenic compounds; (**E**) EMID; and (**F**) Transgenic models. Identically colored boxes represent the same class of animal models, with the first-appeared box designating the model and the later-appeared box being the result of the former, along with the reference citation.

As mentioned, adenomyosis can occur spontaneously in many animal species but typically with a low incidence, which can be enhanced through drug manipulation and surgery. The various existing models are summarized in Figure 2.

Some models are the result of serendipitous discoveries in the endeavor to quest for the result of certain drugs and were found when the object of inquiry was not even the reproductive system itself. As such, while the end result is indeed adenomyosis, the way that the condition was induced may not be consistent with its known or postulated pathogenesis. In other instances, there is no supporting evidence from epidemiological studies. We list these models in Table 1.

It can be argued that for any human disease, the availability of animal models that can faithfully recapitulate the key features of the disease of interest would provide an indispensable tool in the endeavor to unravel the pathogenesis.

Indeed, if one can generate at will an animal model that can recapitulate the major features of the disease, a great deal can be learned of its pathogenesis and pathophysiology from the model. The experimentally induced models offer a consistent, reproducible, and controlled alternative to the spontaneous models and, in many cases, are also much less time-consuming and more economical.

However, an animal disease model that merely possesses the look and feel of its human counterpart may not be really useful in unveiling the pathogenesis of the human condition, even though it may still be helpful in aiding drug research and development. To gain useful insight into the pathogenesis, perhaps one important requirement for an animal model is consistency with epidemiological findings. That is essentially the second requirement for a good theory: that it can explain most, if not all, existing data. In particular, if a theory postulates the cause of adenomyosis, it has to meet the requirements for causality. Of importance, it has to pinpoint the primum movens for the documented aberrations that led to the development of adenomyosis. In this case, while the TIAR theory posits that hyperperistalsis induces microtrauma in the uterus and then adenomyosis, one practical question is what caused the hyperperistalsis in the first place.

Yet in medical research, a good theory cannot just talk the talk. More importantly, it has to walk the walk in order to make an impact on health and disease. To paraphrase Karl Marx, different theories have only explained why or how adenomyosis occurred; the point is to prevent it. This amounts to the third basic requirement for a good theory: it can make useful predictions.

In the following section, we provide a more detailed review of existing models, arranged by possible inducing agents.

#### 3.3.1. Progestogens

Traditionally, it has been held that adenomyosis is more common in women with a history of pregnancy [97], especially if the pregnancy has been carried to, or near, the term [98], suggesting a relationship with exposure to high circulating levels of progesterone. That is, during pregnancy—and even more so in the case of multiple pregnancies—prolonged exposure to progesterone may increase the risk of developing adenomyosis. In all fairness, there are no recent publications on this issue; therefore, its true significance remains to be clarified. Indeed, before or at the time when this review paper was published in the 1980s [97], adenomyosis was diagnosed mostly by histological evaluation after hysterectomy in mostly peri-menopausal women complaining of heavy menstrual bleeding [99]. More recently, thanks to the advances in imaging techniques, early stages of adenomyosis have to be identified in young women of reproductive age [100], even in adolescent girls [101]. Therefore, there is naturally a bias towards more multiparous women using the histology diagnosis criteria. In addition, multiple pregnancies are likely to be associated with an increased risk of abortions, which, in turn, may increase the risk of EMI disruption (EMID) and hence the risk of developing adenomyosis. As such, the role of progesterone as a single causative agent in inducing adenomyosis remains unclear and may be in need of critical reappraisal.

Experimentally, one study, published over a half century ago, found cystic glands penetrated into the myometrium in female Balb/C mice after 12–18 months of chronic and prolonged exposure to progesterone. Synthetic progestins had a similar effect in inducing adenomyosis, although to a less extent than progesterone [81].

A more recent hypothesis of a possible causative role of progesterone involves trophoblast invasion of the inner myometrium during early pregnancy; this may disrupt the EMI, increasing the risk of adenomyosis [102]. In addition, prolonged exposure to progestins may elevate the expression of COX-2 and aromatase in the endometrium [103,104], resulting in an increased local production of estrogens due to the positive-feedback loop linking inflammation and estrogen biosynthesis [105]. The local hyperestrogenism may promote EMT in the endometrium, inducing adenomyosis [106]. The overexpression of COX-2, the gene encoding the rate-limiting enzyme to produce prostaglandin E2 (PGE_2_) and PGF_2α_, could lead to enhanced uterine contractility. Of course, progesterone may act as an anti-inflammatory molecule in myometrium [107], countering the effect of PGE_2_.

#### 3.3.2. Prolactin

The polypeptide hormone prolactin (PRL) is mainly produced and secreted by the pituitary and by the decidua during pregnancy; in addition, an ectopic pituitary graft can increase local PRL levels [108]. Dopamine plays a major role in regulating the PRL secretion by binding to dopamine D2 receptor (DRD2) in both in-situ and grafted pituitary [109,110,111].

In the 1980s, Mori and his colleagues in Japan found that adenomyosis could be induced in some mouse strains by pituitary grafts, either within the uterus or under the renal capsule [83]. Moreover, they found that the incidence of adenomyosis can be reduced by the administration of bromocriptine–mesylate in mice with ectopic pituitary grafts [84]. Bromocriptine is a DRD2 agonist and can suppress the release of PRL [110]. Consistently, SHN mice treated with dopamine antagonists, which resulted in increased PRL release, also developed adenomyosis [112]. Treatment of rats for 90–100 days with fluoxetine hydrochloride, a selective serotonin reuptake inhibitor (SSRI) that can increase the PRL secretion, induced adenomyosis with high incidence in rats [89,113]. In addition, aged female mice deficient in DRD2 developed uterine adenomyosis spontaneously in response to prolonged PRL exposure [90]. Moreover, adenomyotic lesions in mice with induced adenomyosis demonstrated increased PRL receptor (PRLR) expression [114].

Taken together, these observations suggest that high levels of PRL or hyperprolactinism could lead to the development of adenomyosis. However, ovariectomized mice receiving pituitary grafts did not develop adenomyosis [83]. Since ovariectomy effectively removes the major source of estrogens and since estrogen is reported to further increase PRL release in mice with ectopic pituitary graft [115], this seems to suggest an indispensable role of estrogens in PRL-induced adenomyosis.

PRL is a pituitary hormone that has pleiotropic actions on a wide range of tissues [116]. In addition to the pituitary glands, PRL can also be produced by the myometrium, endometrium, and inflammatory cells, which can be modulated by steroid hormones [117] and cytokines [118,119]. The PRLR is expressed in the myometrium and endometrium, suggestive of the functional role of PRL in the uterus [120]. Its actions are likely facilitated by estrogens and progestins [121,122,123]. In particular, PRL is shown to be a mitogen for smooth muscle cells in vitro [124,125,126]. Increased invasiveness of adenomyotic stromal cells, which can be suppressed by the matrix metalloproteinase (MMP) inhibitor, is found in adenomyosis induced by ectopic pituitary grafting in mice [127]. PRL also synergizes with the canonical Wnt/β-catenin signaling pathway via activation of the Notch pathway [128], likely promoting EMT and resulting in the invasion of endometrial epithelial cells into the myometrium, leading to the formation of initial adenomyotic lesions [95,129,130,131,132].

In humans, patients with adenomyosis frequently exhibit hyperprolactinemia [113,133], but one less appreciated result is that the elevated levels of PRL can cause increased pain by promoting nociceptor sensitization through the short isoform of PRLR (RPLR-S) [134,135,136,137,138], the expression of which is also found to be higher in the bovine uterus with adenomyosis [139] and, we can speculate, in humans as well. DRD2 agonists have been shown to suppress PRL-induced adenomyosis and also have therapeutic potential for reducing pain and treating adenomyosis, likely through the promotion of the long isoform of PRLR (PRLR-L) and the inhibition of angiogenesis by DRD2 [140,141,142,143]. It has been shown that following insertion of a vaginal ring containing bromocriptine, women with adenomyosis have alleviated pain, reduced menstrual bleeding, and improved quality of life [144].

Although PRL-induced adenomyosis has been reported in several mouse strains, such as SHN, SLN, Balb/C, C57, C3H, Balb/C and C3H F1 hybrids, C57, and ICR, and also in rats [82,83,84,85], so far there have been no epidemiological data in support for such a link in humans. Whether PRL can be considered as a single causal agent in inducing adenomyosis in humans is unclear.

It is possible that increased PRL levels, due to either ectopic pituitary grafts, administration of SSRI, or DRD2 insufficiency, could cause or exacerbate pain, which subsequently activates the hypothalamus–pituitary–adrenal (HPA) axis, resulting in increased release of catecholamines, which, in turn, may activate adrenoreceptors in the endometrium. The activation of adrenoreceptors in the endometrium may induce EMT or collective cell migration [145,146,147,148], leading to the infiltration of endometrial cells in the myometrium and thus adenomyosis.

### 3.4. Estrogens and Estrogenic Compounds

The reduction of symptoms in postmenopausal adenomyosis patients indicates that adenomyosis is an estrogen-dependent disease [149]. Adenomyosis induced by long-term exposure to estrogens or estrogenic compounds has been documented in several animal models.

The duration of induction varies with animal species. Giving estrogens to mice for more than 10 months can induce adenomyosis, whereas it takes two years for rabbits [66,68]. In ovariectomized adult female rhesus monkeys (*Macaca mulatta*) that received two subcutaneous implants containing 200 mg 17β-estradiol, adenomyosis was found in one out of six at necropsy 16 months later [69]. Postnatal transient estrogen treatment altered the growth factor networks in the uterus of sheep, which had an effect on the structural development of the uterus, including uterine wet weight, thickness of the endometrium, myometrium, and luminal epithelium, and the number of endometrial glands [71]. Consequently, the result of prolonged administration of unopposed estrogen is unpredictable and duration dependent. Long-term exposure to either estrogen or progesterone without the presence of the opposing other hormone increases the risk of the development of adenomyosis [66,81], suggesting that hormonal imbalance may influence the occurrence of the condition.

Recently, Heinosalo et al. found adenomyosis-like phenotype present in transgenic mice overexpressing human 17β-hydroxysteroid dehydrogenase type 1 (HSD17B1); the phenotype appeared at the age of 5.5 months and became more pronounced at 12 months [70] (presented at the 14th World Congress on endometriosis, 6 March 2021). HSD17B1 is highly expressed in human placental syncytiotrophoblast cells throughout pregnancy and in ovarian granulosa cells from primary follicle to corpus luteum stage and is lowly expressed in many other tissues, including endometrium. Therefore, the overexpression of HSD17B1 induces adenomyosis, which is involved in the biosynthesis of estrogens, again highlighting the important role of hyperestrogenism in the development of adenomyosis.

While tamoxifen (TAM) is generally considered to be an anti-estrogen and is used to treat estrogen receptor-positive breast cancer in pre- and post-menopausal patients, its activity actually varies depending on the tissue type [150]. In particular, it exhibits an estrogenic effect in the endometrium [151], and, as such, it is considered to be a member of the selective estrogen receptor modulator (SERM) family [152].

The mouse model of adenomyosis induced by neonatal feeding of tamoxifen is an interesting one [72]; it is strain dependent and also administration route dependent since the injection, instead of feeding of tamoxifen, would not induce adenomyosis [153]. Tamoxifen or toremifene (another SERM) was orally given to neonatal CD-1 mice from 2 to 5 days after birth, and the histogenesis of uterine adenomyosis could be seen on 42–90 days after dosing [72,73,154].

In addition, post-menopausal women with breast cancer who received tamoxifen treatment are reported to have an increased risk of adenomyosis [155]. However, most instances of adenomyosis occur before menopause, and the majority of post-menopausal women do not take tamoxifen. Additionally, there is a vast difference between neonatal and post-menopausal exposure to tamoxifen since the neonatal period is highly sensitive to endocrine-disrupting chemicals [156].

Finally, this animal model also is strain- and even the administration route dependent. When substituted C57 strain mice for CD-1, disordered arrangement of the myometrium rather than adenomyosis took place in the uterine horns [74]. That the success rate of the induction is strain dependent strongly suggests that some particular genetic background may also play a role. Moreover, daily oral administration of tamoxifen failed to induce adenomyosis in female adult rats [157]. More remarkably, neonatal administration of the same dose of tamoxifen to the identical strain of mice via the subcutaneous, rather than the oral, route produces uterine carcinomas rather than adenomyosis [158]. It is thus puzzling as to why the difference in delivery route would lead to a different outcome.

In any case, while the model may still have utility in preclinical studies in drug R&D, such a great variation in outcome according to mouse strains, as well as administration route, raises the doubt as to whether the model can help shed light on the pathogenesis of human adenomyosis.

Disrupted organization of the myometrium may contribute to the adenomyosis caused by tamoxifen, but the exact histological and molecular changes occurring in the neonatal uterus after tamoxifen treatment remain to be explored. Furthermore, PRL has an effect on myometrial smooth muscle cells, but ovariectomized pituitary-grafted C57 mice neonatally fed with tamoxifen do not seem to develop adenomyosis; rather, they show a disordered arrangement of the myometrium. This suggests that disruption of myometrium may be just a necessary, but certainly not a sufficient, condition in the development of adenomyosis. Finally, how this relates to the human condition remains completely unclear.

Diethylstilbestrol (DES), another estrogenic compound, has a structure and function similar to that of tamoxifen. Its teratogenic and carcinogenic effect on the female reproductive tract is well-documented, and adenomyosis is also one of the common lesions in the uterus of mice exposed to DES prenatally [159].

The incidence of DES-induced adenomyosis in mice depends on the dose, administration route, duration, and mouse strain but is generally low. For example, among from 12- to 18-month-old offspring of CD-1 mice exposed to 0.01–100 μg/kg DES on days 9–16 of gestation, only 1 out of 22 (4.5%) in the 5 μg/kg dose group developed adenomyosis [76].

In another experiment, Balb/C and Balb/C-C3H hybrid mice were fed a daily diet containing 0.2 μg/g bodyweight of DES starting from the 7th day after conception until delivery. Adenomyosis-like lesions were found in both strains of mice but with lower incidence in the hybrid strain [75]. It follows that the occurrence of adenomyosis caused by prenatal exposure to DES is sporadic and strain dependent. Its specific mechanism may be related to the effect of DES on the cytogenesis of PRL-producing cells, but unfortunately, there are no supporting epidemiological data [160]. At any rate, long-term, prenatal, or neonatal exposure to DES can no longer be a cause of adenomyosis since the use of DES has been discontinued for decades.

Other estrogenic compounds, such as bisphenol A (BPA), dioxin, and ethinyl estradiol (EE2), have also been shown to induce adenomyosis in different mice strains. Neonatal subcutaneous injection of 10, 100, or 1000 μg/kg/day BPA could induce adenomyosis in CD-1 mice, and the incidence was dose proportional [78]. Adenomyosis developed not only in F1–F2 generations of C57 mice with a history of dioxin exposure but also in F3 generation of C57 mice with an indirect history of dioxin exposure [79]. Exposure to EE2 either prenatally or after sexual maturity could induce adenomyosis in ICR mice, but the highest incidence was seen in mice exposed to EE2 both prenatally and in adulthood [80].

### 3.5. Evidence for More Than One Pathogenesis

It is well-known that the action of estrogen is mediated through estrogen receptors (ERs), which have two isoforms, ERα and ERβ. Both isoforms are expressed in many tissues such as the brain, uterus, ovary, breast, prostate, thymus, spleen, bone, liver, lung, the cardiovascular system, and the gastrointestinal tract [161,162,163,164,165], although the tissue distribution and expression level of ERα and ERβ within the same tissue can be different and even discordant. ERβ typically has a wider tissue distribution than ERα [166]. ERα and ERβ often have incongruent functional characteristics [167,168,169]. ERα promotes while ERβ inhibits proliferation [170], and the activity of ERα can be regulated by ERβ [171,172,173].

TAM can bind with both ERα and ERβ [174] and is used mainly for the treatment of ER-positive breast cancer [175,176]. However, TAM therapy has different efficacy in breast cancer with different ERα and ERβ distribution and has a higher efficacy for breast cancer with higher ERβ expression [177,178]. This seems to suggest that the two receptors have different binding affinities and responsiveness to TAM.

Estrogen and TAM can also activate ER by non-genomic mechanisms, such as through the G-protein coupled receptor 30 (GPR30) signaling pathway. TAM exerts its function as a GPR30 agonist that activates the epidermal growth factor receptor (EGFR) intracellular signaling, MAPK and PI3K/AKT signaling pathways, leading to TAM resistance in breast cancer [179,180,181,182]. Endometrial abnormalities, such as bleeding or endometrial thickness induced by TAM therapy, are also associated with TAM binding to GPR30 [183]. TAM and estrogen can promote cell migration and proliferation by triggering GPR30 activation in endometrial cancer [184,185].

In light of the above, it is conceivable that TAM induces adenomyosis through different ERs, either individually or collectively.

To delineate the roles of specific ERs in TAM-induced adenomyosis in CD-1/ICR mice, we neonatally fed the mice with specific ERα, ERβ, or GPR30 agonists. Specifically, in a manner identical to the TAM-induction of adenomyosis, we neonatally fed the mice with propylpyrazoletriol (PPT, an ERα agonist), diarylpropionitrile (DPN, an ERβ agonist), and G-1 (a GPR30 agonist). Remarkably, neonatal feeding of PPT or G-1 did not result in adenomyosis, but the neonatal feeding of 5 mg/kg DPN yielded adenomyosis in 50% of ICR mice [77]. Of particular note, all adenomyotic lesions were restricted to the subserosal layer reminiscent of extrinsic/external adenomyosis in humans [186,187] and quite different from those induced by TAM. These findings not only demonstrate that TAM does not induce adenomyosis through a single ER but also suggest that the pathogenesis of extrinsic/external adenomyosis may be different from other subtypes of adenomyosis. The somewhat lower incidence of adenomyosis following neonatal feeding of DPN (50% vs. 100% in TAM-induced adenomyosis) is likely due to the lower blood concentration resulting from the oral administration. Indeed, the plasma DPN concentration through oral administration is significantly lower than that of intramuscular or subcutaneous injection [188]; hence, there is room for optimization of this animal model by changing the route of administration or increasing the oral dosage to increase the incidence of adenomyosis induced by DPN.

This experiment, if further validated, is perhaps the best piece of evidence suggesting that different subtypes of adenomyosis may have different pathogenesis.

### 3.6. In Utero Exposure to Exogenous Estrogens

From Figure 2C,D, it can be seen that prenatal exposure to estrogens or estrogenic compounds induces adenomyosis in several animal models. This suggests that exposure during a very sensitive time period in the development can substantially increase the risk of developing adenomyosis in later life. This seems to echo the finding that shorter anogenital distance (AGD), a sign of higher estrogen levels in utero, is associated with the risk of developing endometriosis [189,190,191,192,193]. Our own data also show that shorter AGD is associated with a higher risk of developing adenomyosis (Ding et al., unpublished data). Taken together, these data strongly suggest that in utero exposure to higher levels of estrogens increases the risk of developing adenomyosis. Indeed, several studies have reported that prenatal exposure to estrogen or estrogenic agents causes disruption in the EMI [76,194,195,196], which may provide a hotbed for the genesis of adenomyotic lesions.

### 3.7. Endometrial–Myometrial Interface Disruption

While it is generally recognized that adenomyosis is induced by endometrial epithelial and stromal cells invasion into the myometrium through EMT [106,197], how exactly the endometrium invades, in a seemingly unimpeded manner, the myometrium wall is a puzzling conundrum. In addition, why this invasion happens only in a small proportion of women, but not in their majority, is also completely unclear.

The endometrium, which can be further divided into *functionalis* and *basalis* layers, is directly in contact with the EMI. Conceivably, structurally weakened myometrium because of repeated trauma to the EMI may facilitate the invagination or the invasion of endometrial cells into the EMI and then to the myometrium. A large body of epidemiological data has shown, quite consistently, that iatrogenic uterine procedures, such as dilatation and curettage (D&C) and induced abortion, increase the risk of developing adenomyosis later in life [133,198,199,200,201,202].

Leyendecker’s TIAR theory hinges critically on the feed-forward loop linking inflammation and estrogen production [36], which seems the case for ectopic endometrium [203], but is not necessarily true for eutopic endometrium. For this reason, in light of ample epidemiological data linking iatrogenic uterine procedures and adenomyosis, we proposed a new hypothesis on the pathogenesis of adenomyosis, termed EMI disruption (EMID) due to physical damage or trauma [39]. To test this hypothesis, we induced EMID by mechanical injury as well as thermal injury [91]. We found that 100% of Balb/C mice and 83.3% of C57 mice experiencing mechanically induced EMID developed adenomyosis 3 months after the procedure.

It is worth mentioning that adenomyosis induced by pituitary transplantation may also be caused, at least in part, by injury during the operation. Depending on the severity of thermally induced EMID, the incidence of adenomyosis was found to range between 30% and 66.7%. More remarkably, perioperative intervention by administration of either a β-blocker or aprepitant, a neurokinin receptor 1 (NK1R) inhibitor, significantly reduced the risk of developing adenomyosis [91].

The EMID hypothesis and its supporting experimental data strongly suggest that iatrogenic uterine procedures increase the risk of developing adenomyosis, and the risk is proportional to the extent and severity of injury to the EMI or the amount and severity of EMID. The risk is also dependent on the mode of injury, for example, mechanical or thermal. These experiments not only provide evidence in support of the EMID hypothesis but also establish an easy and economical animal model for adenomyosis. More importantly, in light of the ubiquity of uterine procedures nowadays, the EMID hypothesis suggests an interventional procedure to mitigate the risk of developing adenomyosis espoused by these procedures. Furthermore, the insight to be gained from EMID-induced adenomyosis may help to unravel its pathogenesis due to other causes.

### 3.8. Other Models

Adenomyosis has also been found in the uterine horns of some transgenic mice, such as Dicer inactivated mutant mice, follicular stimulating hormone (FSH) receptor (FSHR)-haplo-insufficient mice, Foxl2 deleted mice, and mice with impaired prostaglandin D2 (PGD_2_) synthesis [92,93,94,204]. Incidentally, the FSHR polymorphism has been reported to be associated with the risk of developing endometriosis and the fertility status of patients [205,206]. PGD_2_ is mainly involved in myometrial contraction [96], and the peristalsis of myometrium activated by hyperestrogenism may increase the risk of developing adenomyosis based on the notion of TIAR. Deregulated Wnt signaling pathways play a crucial role in the development of adenomyosis in the Foxl2 deleted mice, and constitutive activation of β-catenin in the murine uterine horns can also lead to the development of adenomyosis through promoting EMT in the epithelial cells [95]. However, since so far there has been no report on the association between adenomyosis and genetic mutations or polymorphisms on either Dicer, Wnt, β-catenin, or Foxl2, the utility of these models in elucidating the pathogenesis of adenomyosis appears to be limited.

### 3.9. The Root Causes for Pathogenesis

Animal models of the disease that are congruent with epidemiological data stand a good chance to unravel the disease pathogenesis. Using this standard, few existing animal models of adenomyosis pass the test.

**Figure 3:**
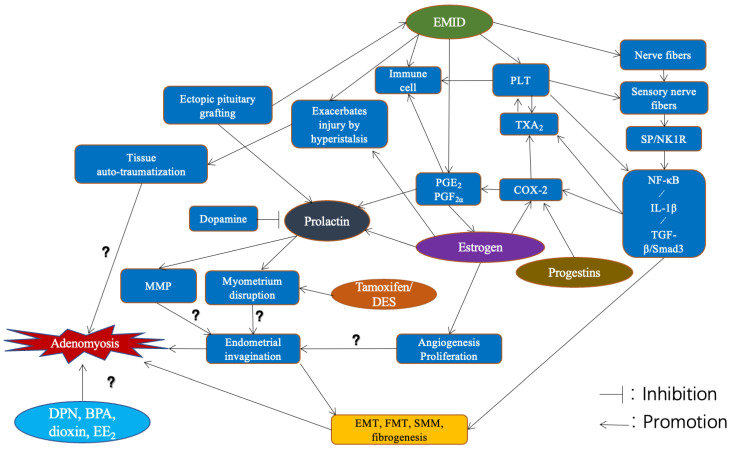
Schematic illustration of different animal models of adenomyosis, along with their possible mechanisms. Abbreviations used: SP—substance P; TXA_2_—thromboxane A_2_; COX-2—cyclooxygenase-2; PGE_2_—prostaglandin E_2_; PGF_2α_—prostaglandin F_2α_; TGF-β1—transforming growth factor β1; EMID—endometrial–myometrial interface disruption; MMP—matrix metalloprotein; NK1R—neurokinin 1 receptor; EMT—epithelial–mesenchymal transition; FMT—fibroblast–myofibroblast transition; SMM—smooth muscle metaplasia.

Laboratory mice are the most frequently used experimental animal models to investigate the pathogenesis of adenomyosis due to their economy, ease of generation and maintenance, and relatively high success rate of induction. However, as reviewed above, the diversity of induction methods and the difference in incidence due to different dosages and mouse strains underscore the complexity and likely multiple pathogeneses of adenomyosis (Figure 3). In fact, several possible culprits in the genesis of adenomyotic lesions can be identified: aberrant production of sex steroid hormones; changes in a uterine microenvironment chronically exposed to estrogens or estrogen-like compounds, especially during the critical developmental periods such as pre- or peri-neonatal period; dysfunctional contractility of the EMI, physical trauma to the EMI, neural involvement, and aberrant immune response.

As of now, the only animal model that seems to be consistent with epidemiological evidence is EMID-induced adenomyosis [91]. However, EMID is unlikely to be the only cause for the condition, simply because many patients who have adenomyosis did not have any history of uterine procedures.

The search for the pathogenesis of adenomyosis essentially consists in finding the cause(s) for the disease. As such, animal models that can help us to shed light on the pathogenesis should, ideally, satisfy the nine criteria for causality proposed by Austin Bradford Hill [33], which are association strength, consistency, biological gradient, specificity, temporality, biological plausibility, experimental evidence, analogy, and coherence [207] (Table 2). Using Hill’s criteria as a bar, all animal models of adenomyosis appear to satisfy, by default, the criteria of experimental evidence and temporality, and some may satisfy the criterion of biological gradient (Table 3). However, many fail to meet the criteria of consistency (say, strain- or delivery route dependency) and coherence (inconsistent with or lack of support by epidemiological data). Some are questionable in the category of association strength (as manifested by low incidence). The EMID model [91] appears to satisfy all criteria.

## 4. Discussion

### 4.1. Pathogenesis and Beyond

Just as endometriosis [54,208], adenomyosis also has several subtypes and appears to be pathogenetically and phenotypically heterogeneous. Our own data show that neonatal feeding of ERβ-agonist-induced adenomyosis is seemingly different from that induced with TAM and is similar to the extrinsic/external adenomyosis in humans. This suggests that, at the very least, the extrinsic/external adenomyosis may be pathogenetically different from other subtypes of adenomyosis.

In fact, in an MRI classification of adenomyosis, Kishi et al. have noted that patients with intrinsic/internal adenomyosis, i.e., adenomyotic lesions that are close to the endometrium, are more likely to have a history of curettage as compared with those other subtypes of adenomyosis (32.2% vs. up to 9.1%) [187]. Consistently, focal adenomyosis of the outer myometrium or external/extrinsic adenomyosis is found to be closely linked with deep endometriosis [45,49,50], raising the possibility that some subtypes of adenomyosis and deep endometriosis may be two forms of the same disease [50].

Recent studies also indicate that different subtypes of adenomyosis have different symptomology. For example, internal adenomyosis (that is, lesions proximal to the endometrium) is more likely to be associated with heavy menstrual bleeding, while external adenomyosis (that is distal to the endometrium but proximal to the uterine serosa) is closely linked with deep endometriosis [47], a finding that has also been mentioned in Kishi et al. [187]. Indeed, the recent discovery of the three-dimensional histoarchitecture of the endometrium [209,210] would strongly support the notion that EMID resulting from iatrogenic uterine procedures, such as curettage, would likely cause intrinsic/internal, but less likely extrinsic/external, adenomyosis.

The recognition of this heterogeneity may help us better understand the pathogenesis and pathophysiology of adenomyosis. This also would call for better phenotyping in future epidemiological studies. Failure to distinguish different subtypes of adenomyosis that are likely to be pathogenetically and phenotypically different may diminish the signal-to-noise ratio, leading to erroneous conclusions. Better phenotyping of adenomyosis may also help to reconnect the seemingly disconnect between theories of adenomyosis pathogenesis and epidemiological studies in search of risk factors for adenomyosis.

The EMID hypothesis can be proven experimentally and can explain why iatrogenic uterine procedures increase the risk of developing adenomyosis. In addition, the hypothesis would predict that the magnitude of the risk of developing iatrogenically induced adenomyosis may depend not only on the mode but also on the extent and severity of EMID [91]. This may explain why not all women who underwent iatrogenic uterine procedures develop adenomyosis since different women may simply experience different modes or degrees of EMID. If EMID is extensive and severe enough, there should be an increased risk of developing adenomyosis as opposed to minor or no EMID. More importantly, the EMID, as a tissue trauma, would cause tissue injury, eliciting the release of substance P [211] and PGE_2_ [212], and activate the hypothalamic–pituitary–adrenal (HPA) axis, resulting in increased release of catecholamines such as adrenaline/ noradrenaline, which, in turn, may suppress cell-mediated immunity [213]. The abundance of adrenergic nerve fibers in the uterus [214,215] seems to give credence to this view. This would indicate that perioperative intervention to counter either the action of the receptor for substance P, neurokinin receptor 1 (NK1R), or to contain the release of PGE_2_ by inhibiting COX-2, or the adrenaline receptor would reduce the risk of adenomyosis resulting from EMID [91]. Indeed, perioperative administration of an NK1R inhibitor or a β-blocker plus a COX-2 inhibitor did reduce the risk of developing adenomyosis in mice [91].

### 4.2. Knowledge Gaps

Although the EMID hypothesis is now supported by experimental evidence, there is still a vast knowledge gap. For example, what is the exact molecular mechanism underlying EMID-induced adenomyosis? How does the perioperative intervention reduce the risk of developing adenomyosis? What is its underlying mechanisms of action? Can the benefits of a perioperative intervention be translated into a clinical setting? Given the popularity of iatrogenic uterine procedures nowadays and given the excellent safety profiles of β-blockers, andrographolide, and NK1R inhibitors such as aprepitant, it seems that clinical studies or trials are badly needed.

More fundamentally, recent advancement in delineating the three-dimensional histoarchitecture of the human endometrium has revealed that the human endometrial glands have a unique and complex 3-D structure that is drastically different from the traditional view [209,210]. In essence, the seemingly non-branching, single, vertical *functionalis* glands originate from a complex horizontally interconnecting network of *basalis* glands [209], which do not detach during menstruation [216] and are believed to harbor endometrial epithelial stem cells. Since the *basalis* glands are physically located at the EMI region, any EMID event is likely to disrupt these glands. Hence, it is likely that the EMID procedure would cause injury to *basalis* glands, inaugurating a chain of events of tissue repair, such as platelet aggregation, recruitment of immune cells, release of inflammatory cytokines, hypoxia, and increased local production of estrogen, that collectively lead to the infiltration of glandular epithelial cells with possible pluripotent capability into the myometrium, establishing the adenomyotic lesion [39]. The finding that adenomyosis lesions are stereoscopically characterized by an “ant colony-like network” that directly connects with endometrial glands [210] appears to support this notion. Yet, how the *basalis* glands are involved in the formation of initial lesions and whether or not there are other aiders and abettors would await future investigations. Here, it may be helpful not to be fixated on a few pre-determined suspecting factors but, rather, to keep an open mind, cast a wider net whenever possible, and be practical.

In addition, while hyperprolactinemia is common in patients with adenomyosis [113,133], the exact role of PRL in inducing the condition has not been fully characterized. In particular, given the diverse functions of PRLR-S and PRLR-L, it may be time to evaluate their presence, abundance, and functions in human adenomyosis.

Moreover, given the shorter AGD in women with adenomyosis (Ding et al., unpublished data) similar to those with endometriosis [189,190,191,192,193], it seems that the in-utero exposure to elevated levels of estrogen may increase the risk of developing adenomyosis. How and why this is the case would warrant further confirmation and investigation.

The recently documented close link between external/extrinsic adenomyosis and deep endometriosis [45,46,47,48,49,50] strongly suggests that the two disease entities may have a causal relationship. It is possible that the two entities may represent the same disease [50]. Alternatively, one disease entity could be simply caused by the other due to physical proximity and the invasiveness of ectopic endometrium [217]. Very limited mutation frequency data seem to indicate that deep endometriotic lesions concurrent with adenomyosis had a higher KRAS mutation frequency than neighboring adenomyotic lesions (Figure 4 in [218]), and, as such, suggest that external/extrinsic adenomyotic lesions may be secondary to and colonized by neighboring deep endometriotic lesions. Unfortunately, these data are too scarce to be conclusive. In principle, for these two disease entities, one can simply resolve the issues of which one was the first established and of whether the two have any relationship by constructing a phylogenetic relationship between the two through the use of the molecular clock. Recent methodological advancements such as the use of the DNA methylation clock [219] should be able to help determine whether there is any relationship between the two disease entities as well as which entity existed first.

## 5. Conclusions

So far, several hypotheses/theories on the pathogenesis of adenomyosis have been proposed and articulated, and understandably, the focus is often on their novelty, ingenuity, and explanatory power. However, the aspects of falsifiability and of their predictive power are frequently overlooked. This is unfortunate since if a theory cannot be falsifiable, then it is akin to a religion, and if the theory cannot be used to make useful predictions, then its utility is questionable or perhaps dubious. Arguably, falsifiability and predicting power are equally important as novelty, ingenuity, and explanatory power to a good theory.

To prove or refute a hypothesis or even a theory on the pathogenesis of adenomyosis, animal models are arguably indispensable and critical. This is due not only to the logistic and ethical constraints on human experimentation but also to the fact that in many animal species, including rodents, adenomyosis can occur spontaneously. Even if a prospective, longitudinal human study to test a specific hypothesis of adenomyosis pathogenesis can be launched, animal models would still be needed to tease out the underlying molecular mechanisms. The ease of manipulating the environment, the economy, and the shorter timespan that an animal model can offer would also help to generate new hypotheses to be tested in future epidemiological studies. In this sense, animal models of adenomyosis are here to stay.

The ultimate goals for establishing animal models of adenomyosis that are consistent with epidemiological data are to help unveil its pathogenesis, to translate experimental data into better diagnosis and better management of the disease, and, hopefully, to devise interventional measures to prevent the disease. The EMID mouse model has helped us to unveil, at least in part, some possible pathogenetic causes for developing adenomyosis. However, EMID is not the sole cause for adenomyosis. Future studies, including animal studies, are warranted to understand how and why in utero and/or prenatal exposure to elevated levels of estrogens increases the risk of developing adenomyosis in adulthood, to elucidate whether PRL plays any role in the pathogenesis of adenomyosis, and to identify sufficient condition(s) that cause adenomyosis.

Finally, there is still a glaring disconnect between theories of pathogenesis and epidemiological findings. Part of this disconnect may stem from the lack of appreciation that there are different subtypes of adenomyosis, which may have different pathogenesis. In future investigations, perhaps a clearer definition of adenomyosis subtypes and better phenotyping may help to reconnect pathogenetic theories and epidemiological findings.

## Figures and Tables

**Table 1:** Summary of animal adenomyosis models by species, strain, induction method, duration of induction, and results.

Pathogenesis		Species/Strain	Induction Method	Duration of Induction	Outcome	References
Estrogen or estrogenic compounds	Estrogen	AB/Jena and DBA 2/Jena hybrid mouse	Pregnant F1 animals were orally given 1 mg/kg of 17β-phenylaminocarbonyloxyestra-1,3,5(10)-triene-3-methyl ether daily on days 12 to 16 post coitus	≥10 months	Adenomyosis was found in 10 out of 27 virgin female offspring of estrogen-treated dams from 16 to 33 months of age	[66,67]
		Rabbit	Stilbestrol (5 mg/mL) was injected i.m.	2 years	Adenomyosis	[68]
		Rhesus monkeys (*Macaca mulatta*)	S.c. implants containing 200 mg estradiol	16 months	Adenomyosis	[69]
		Transgenic mouse	Overexpressing human HSD17B1	5–12 months	Adenomyosis appeared at the age of 5.5 months and became more severe at 12 months	[70]
		Sheep	Postnatal daily i.m. injections of estradiol-17β benzoate at a dose of either 0, 0.01, 0.1, 1, or 10 μg/kg body weight from PND 14–27 (period one) or PND 42–55 (period two)	PND 28, PND 56, PND 112	Immediate responses to EB treatment included dose- and age-dependent increases in uterine wet weight, thickness of the endometrium, myometrium, and LE, but decreases in endometrial glands on PND 28 and 56. Transient exposure to EB decreased gland number and thickness of the endometrium and LE on PND 112	[71]
	Tamoxifen	CD-1 mouse	Tamoxifen, toremifene, and raloxifene dosed orally 2–5 days after birth consecutively	42–90 days	Uterine adenomyosis was found in all (14 out of 14) mice dosed with tamoxifen and most mice (12 out of 14) treated with toremifene, in only one animal treated with raloxifene	[72,73]
		C57 mouse	Female C57/BL6J pups (*n* = 20) were treated with oral tamoxifen (1 mg/kg) from age 1 to 5 days	5, 10, 15, and 42 days of age	Causes disruption of myometrial development but not adenomyosis	[74]
	Diethylstilbestrol	Balb/c or Balb/c and C3H inbred mouse	Pregnant mice were fed a diet containing 0.2 μg/g (of bodyweight) of DES continuously on the seventh day of pregnancy until the morning after delivery of the young	18 months of age	Resembled adenomyosis occurred in Balb/c mice with the lesser frequency encountered in the hybrid strain	[75]
		CD-1 mouse	Pregnant outbred mice were treated s.c. with daily doses of DES ranging from 0.01 to 100 jug/kg on days 9 to 16 of gestation	12 to 18 months of age	1/22 adenomyosis in 5 ug/kg group	[76]
	Diarylpropionitrile (DPN)	ICR mouse	Mice in DPN group were dosed orally with 5 mg/kg DPN from day 2 to day 5 after birth	3 months	Neonatal feeding of DPN resulted in adenomyosis in 50% of the mice, but the adenomyotic lesions were located exclusively near the serosa	[77]
	Bisphenol A (BPA)	CD-1 mouse	Outbred female CD-1 mice were treated on days 1–5 with subcutaneous injections of BPA (10, 100, or 1000 μg/kg/day) dissolved in corn oil or corn oil alone (Control)	18 months	Adenomyosis occurred in all groups with an increasing trend in the two highest BPA groups (6% (1/18) Controls, 9% (2/23) BPA-10, 20% (4/20) BPA-100, and 19% (3/16) BPA-1000)	[78]
	Dioxin	C57 mouse	Pregnant mice (F0) were exposed to dioxin (10 μg/kg) in corn oil or vehicle alone by gavage on E15.5 (when organogenesis is complete)	10–12 weeks	Adenomyosis was identified in most animals with a history of direct (F1–F2) or indirect (F3) dioxin exposure. However, although 70% (*n* = 10) of F1 animals exhibited deep adenomyosis, the incidence of advanced disease was slightly lower in F2 mice (63%; *n* = 11) and F3 animals (56%; *n* = 9)	[79]
	Ethinyl estradiol (EE2)	ICR mouse	Pregnant mice were exposed to 0.01 mg ethinyl estradiol (EE2)/kg per day or vehicle (olive oil) through oral intubation from day 11 to 17 of gestation. They delivered their offspring and raised them. When the experimental female F1 mice were at 8 weeks of age, they were not exposed to EE2 or to the same dose of EE2 or to vehicle twice a week until 20 weeks of age	28 weeks	These findings indicate that adenomyosis is induced through either exposure to EE2 prenatally or after sexual maturity, but the highest frequency is seen through the combined exposures	[80]
Progesterone		Balb/c mouse	S.c. implantation of pellets	12–18 months	Present in almost all animals receiving 665 or 900 µg/day	[80,81]
Prolactin	Pituitary grafts	SHN and SLN mouse	Ectopic (intrauterine and under the renal capsule) pituitary transplantation	90 days	Incidence: 100%	[82,83]
		Balb/c mouse	Anterior pituitary (AP) isografting at 8 weeks of age	36 weeks	Increased the incidence of adenomyosis in mice	[84]
		Wistar rat	Transplantation of a single anterior pituitary gland into the uterine lumen	12 months	Adenomyosis was induced in six out of eight Wistar rats	[85]
		Balb/c mouse, C3H mouse, or Balb/c and C3H F1 hybrids	Transplantation of pituitary into the mammary tissue	6 months	Lesions of adenomyosis were frequent in uteri of C3H and F1 hybrids but essentially absent from Balb/c animals	[86]
		Balb/c, C57, C3H mouse	Pituitary was transplanted into the uterine cavity	20 weeks	Adenomyosis had formed in the uteri of 22 (91.7%) mice out of 24 Balb/c mice after the transplantation of pituitary glands. Similar findings were obtained by experiments with C3H and C57 mice	[87]
	Dopamine antagonists	SHN mouse	SHN female mice were subcutaneously injected with dopamine antagonists for 30 days or 50 days.	70 or 90 days of age	The incidences of adenomyosis in the experimental groups of mice for 50 days rose up to over 70%	[88]
	Fluoxetine	Wistar rat	2 mg/kg fluoxetine were given to rats by gavage	98 days	Histological studies revealed 11 cases of adenomyosis in the noncastrated group receiving fluoxetine	[89]
	Transgenic mouse	Dopamine D2 receptor (DRD2)-deficient mouse	Mice that are deficient in functional D2 receptors were generated	One year old	A large proportion of the female DRD2 deficient mice developed uterine adenomyosis, most commonly in mice greater than one year of age	[90]
Endometrial–myometrial interface disruption (EMID)		Balb/c and C57 mouse	Mechanically induced EMID or thermally induced EMID	8–12 weeks	Adenomyosis developed in the majority of mice in the EMID groups (83.3% in C57BL/6 mice, 100% in Balb/c mice); adenomyosis was found in 66.7% of the EMID mice 10 weeks later	[91]
Other transgenic models	Dicer	Dicer inactivated mutant mice	Dicer was inactivated in Müllerian duct mesenchyme-derived tissues of the reproductive tract of the mouse, using an Amhr2-Cre allele	>4 months of age	The glands were found within the myometrium.	[92]
	FSHR	FSH receptor-haplo insufficient mice	The animals of the required genotype were produced by breeding 129T2/SV EmsJ Fshr^−/−^ male and females of 3–5 months	12 months of age	Some uteri showed endometrial glands deeply penetrating the myometrium	[93]
	Foxl2	Foxl2 deleted mice	Conditional deletion of Foxl2 in the PN uterus using PR-Cre ^(Pgrcre/+)^ mice	PN15, PN25, adult	Myometrial disorder	[94]
	β-catenin	Conditionally stabilized β-catenin mouse	Mice that expressed a dominant stabilized β-catenin in the uterus were used by crossing PR-Cre mice with Ctnnb1^f(ex3)/+^ mice	4 months of age	The incidence of 40% at 4 months of age and 80% at 6 months of age	[95]
	PGD2	PGD2 synthesis impaired mice	PGD2 is not produced due to invalidation of both lipocalin hematopoietic type (L-PGDS and H-PGDS) genes	6 months of age	HE staining showed the presence of focal adenomyosis in 35% (*n* = 9 from 28) of knockout mice	[96]

Abbreviations: DES—diethylstilbestrol; i.m.—intramuscular, intramuscularly; s.c.—subcutaneous; PN—postnatal; PND—postnatal day; PR—progesterone receptor; DPN—diarylpropionitrile.

**Table 2:** The Austin Bradford Hill criteria for causality. Adapted from Hill [33], following [207].

Criterion	Comment
Strength of association (Sa)	If the relative risk is “strong”, there is less likelihood that there are other adequate explanations for the observed association.
Consistency (Cs)	Is the association consistent over the various studies?
Biological gradient (Bg)	Is there an exposure–response relationship exhibited over the range of studies?
Specificity (Sp)	Is the association limited to a particular outcome?
Temporality (Tm)	Does the exposure precede the outcome?
Biological plausibility (Bp)	Is the proposed association explained by a biologically plausible mechanism?
Experimental evidence (Ee)	Are there experimental studies that support the association?
Analogy (An)	Is the proposed causal relationship analogous to some other accepted cause and effect?
Coherence (Ch)	Does the proposed relationship seriously conflict with generally known facts about the natural history and biology of the disease?

**Table 3:** A summary of epidemiological data in support for animal models of adenomyosis and which criterion of Austin Bradford Hill’s criteria for establishing causality are satisfied.

Induction Agent	Evidence in Humans?	References	Which Hill’s Criterion or Criteria Are Satisfied
Estrogen	No direct support	[149,189,190,191,192,193]	Bg, Tm, Bp, Ee, An
Tamoxifen	No	No	As, Bg, Tm, Ee, An
Diethylstilbestrol (DES)	No	No	Tm, Ee, An
Diarylpropionitrile (DPN) Bisphenol A (BPA) Dioxin Ethinyl estradiol (EE2)	No	No	Tm Ee
Progestins	No direct support	[97]	Sa, Tm, Bp, Ee, An
Prolactin	No direct support	[133,144]	Sa, Cs, Tm, Bp, Ee, An
Fluoxetine	No	No	Sa, Tm, Ee, An
Endometrial–myometrial interface disruption (EMID)	Yes	[133,198,199,200,201,202]	Sa, Cs, Bg, Sp, Tm, Bp, Ee, An, Ch
Other models	No	No	Sa, Cs, Sp, Tm, Bp, Ee, An for the conditionally stabilized β-catenin mouse Tm, Ee for the others

## Data Availability

All data generated or analyzed are included in the results of the manuscript.

## Abbreviations

AGD	anogenital distance
DES	diethylstilbestrol
DPN	diarylpropionitrile
DRD2	dopamine D2 receptor
D&C	dilatation and curettage
EMI	endometrial–myometrial interface
EMID	endometrial–myometrial interface disruption
EMT	epithelial–mesenchymal transition
FMT	fibroblast-to-myofibroblast transdifferentiation
FSH	follicular stimulating hormone
FSHR	follicular stimulating hormone receptor
HPA	hypothalamus–pituitary–adrenal
HSD17B1	17β-hydroxysteroid dehydrogenase type 1
MMP	matrix metalloproteinase
NK1R	neurokinin receptor 1
PGE_2_	prostaglandin E2
PGF_2α_	prostaglandin F2α
PPT	propylpyrazoletriol
PRL	prolactin
PRLR	prolactin receptor
ReTIAR	repeated tissue injury and repair
SERM	selective estrogen receptor modulator
SMM	smooth muscle metaplasia
SSRI	selective serotonin reuptake inhibitor
TIAR	tissue injury and repair
